# Safety of alemtuzumab in a nationwide cohort of Finnish multiple sclerosis patients

**DOI:** 10.1007/s00415-021-10664-w

**Published:** 2021-07-13

**Authors:** Ilkka Rauma, Tiina Mustonen, Juha Matti Seppä, Maritta Ukkonen, Marianne Männikkö, Auli Verkkoniemi-Ahola, Marge Kartau, Jukka T. Saarinen, Liisa Luostarinen, Sakari Simula, Mervi Ryytty, Riitta Ahmasalo, Jussi O. T. Sipilä, Ilkka Pieninkeroinen, Tero Tapiola, Anne M. Remes, Hanna Kuusisto

**Affiliations:** 1 Faculty of Medicine and Health Technology, Tampere University, Tampere, Finland; 2 Department of Neurology, Tampere University Hospital, Tampere, Finland; 3 Department of Neurology, Seinäjoki Central Hospital, Seinäjoki, Finland; 4 Neuro Center, Kuopio University Hospital, Kuopio, Finland; 5 Department of Neurology, Satasairaala, Pori, Finland; 6 Clinical Neurosciences, Neurology, Helsinki University Hospital and Helsinki University, Helsinki, Finland; 7 Department of Neurology, Vaasa Central Hospital, Vaasa, Finland; 8 Department of Neurology, Päijät-Häme Central Hospital, Lahti, Finland; 9 Department of Neurology, Mikkeli Central Hospital, Mikkeli, Finland; 10 Medical Research Center, Oulu University Hospital, Oulu, Finland; 11 Research Unit of Clinical Neuroscience, University of Oulu, Faculty of Medicine, Oulu, Finland; 12 Department of Neurology, Lapland Central Hospital, Rovaniemi, Finland; 13 Department of Neurology, North Karelia Central Hospital, Siun Sote, Joensuu, Finland; 14 Department of Clinical Neurosciences, University of Turku, Turku, Finland; 15 Department of Neurology, Kymenlaakso Central Hospital, Kotka, Finland; 16 Department of Neurology, South Karelia Central Hospital, Lappeenranta, Finland; 17 Department of Health and Social Management, University of Eastern Finland, Kuopio, Finland; 18 Department of Neurology, Kanta-Häme Central Hospital, Hämeenlinna, Finland

**Keywords:** Alemtuzumab, Autoimmunity, Drug-related side effects and adverse reactions, Incidence, Multiple sclerosis, Safety

## Abstract

**Background:**

Alemtuzumab is an effective disease-modifying therapy (DMT) for highly active multiple sclerosis (MS). However, safety concerns limit its use in clinical practice.

**Objectives:**

To evaluate the safety of alemtuzumab in a nationwide cohort of Finnish MS patients.

**Methods:**

In this retrospective case series study, we analyzed the data of all but two MS patients who had received alemtuzumab in Finland until 2019. Data were systematically collected from patient files.

**Results:**

Altogether 121 patients were identified, most of whom had received previous DMTs (82.6%). Median follow-up time after treatment initiation was 30.3 months and exceeded 24 months in 78 patients. Infusion-associated reactions (IARs) were observed in 84.3%, 57.3%, and 57.1% of patients during alemtuzumab courses 1–3, respectively. Serious adverse events (SAEs) were observed in 32.2% of patients, serious IARs in 12.4% of patients, and SAEs other than IARs in 23.1% of patients. Autoimmune adverse events were observed in 30.6% of patients. One patient died of hemophagocytic lymphohistiocytosis, and one patient died of pneumonia. A previously unreported case of thrombotic thrombocytopenic purpura was documented.

**Conclusions:**

SAEs were more frequent in the present cohort than in previous studies. Even though alemtuzumab is a highly effective therapy for MS, vigorous monitoring with a long enough follow-up time is advised.

## Introduction

Alemtuzumab (Lemtrada) is a humanized monoclonal antibody against the cell surface antigen CD52. The efficacy of alemtuzumab when compared to subcutaneous interferon β-1a for relapsing–remitting multiple sclerosis (RRMS) was initially demonstrated in three randomized clinical trials [1–3]. The extension studies of these core trials are currently providing safety and efficacy data from 5 to 12 years of follow-up [4–7].

The therapeutic effect of alemtuzumab is thought to be mediated by depletion of circulating T- and B-lymphocytes, followed by a distinct pattern of lymphocyte repopulation [1, 8–10]. There is also evidence that alemtuzumab may have remodeling effects on the innate immune compartment [11]. Alemtuzumab is considered to be the first immune reconstitution therapy for RRMS.

Unfortunately, the use of alemtuzumab is limited by various safety concerns. As with most infused biological therapies, infusion-associated reactions (IARs) may occur. The most common signs or symptoms of an IAR after alemtuzumab are headache, rash, pyrexia, nausea, and flushing [12]. The symptoms are generally manageable with a pretreatment protocol consisting of intravenous steroid infusions on the first three days of any course of alemtuzumab, and additional antihistamine or antipyretic treatment administered at the physician’s discretion [12, 13].

As alemtuzumab profoundly affects the immune system, opportunistic infections may occur [1–3]. However, the incidence of infections declines after the first year and does not increase with successive courses of alemtuzumab [4, 5]. In addition to the commonly occurring herpetic infections, less frequent pathogens such as *Listeria monocytogenes* have been reported [14]. To prevent herpetic infections, prophylactic oral acyclovir is used daily during the infusions and for one month thereafter [13].

A special safety concern of alemtuzumab revolves around its association with the development of secondary autoimmune diseases, such as thyroid diseases, immune thrombocytopenic purpura (ITP), and autoimmune nephropathy [2, 3]. The mechanism of secondary autoimmunity is not entirely understood. It has been suggested that the B-lymphocyte depletion and repopulation in the absence of T-lymphocyte regulation is a key factor in the development of secondary autoimmunity in genetically susceptible individuals [8]. Furthermore, an overproduction of IL-21 due to genetic factors has been suspected to predispose to alemtuzumab-induced autoimmunity [15]. However, repopulation kinetics of the evaluated peripheral lymphocyte subsets do not predict autoimmune adverse event (AE) occurrence, and biomarkers that would predict risk for autoimmune events have not been identified [16].

Reports of various new AEs have been published in recent years, including acute acalculous cholecystitis (AAC), acute coronary syndrome, autoimmune hepatitis, and hemophagocytic lymphohistiocytosis (HLH) [17–20]. In 2019, the European Medicines Agency (EMA) restricted the use of alemtuzumab for RRMS and initiated a review due to serious autoimmune and cardiovascular AEs [21]. The final decision of the European Commission was issued in 2020 [22]. According to the new recommendations, the drug should only be used for RRMS if the disease is highly active despite treatment with at least one other disease-modifying therapy (DMT), or if the disease is worsening rapidly. Furthermore, new contraindications were introduced, including concomitant autoimmune diseases other than multiple sclerosis (MS) [22].

Finland is a genetically isolated Nordic country with a high incidence of MS as well as other autoimmune diseases such as type 1 diabetes (T1D) and coeliac disease [23–27]. Therefore, we hypothesized that the safety profile of alemtuzumab for MS might differ from previous reports. Remarkably, the World Health Organization (WHO) initiated the third Global Patient Safety Challenge in 2017 aiming to reduce the global level of severe, avoidable medication-related harm by 50% in the next five years [28]. Our nationwide study of Finnish MS patients, working towards the same goal, evaluated the safety of alemtuzumab treatment in a genetically isolated population.

## Materials and methods

### Patients

**Fig. 1:**
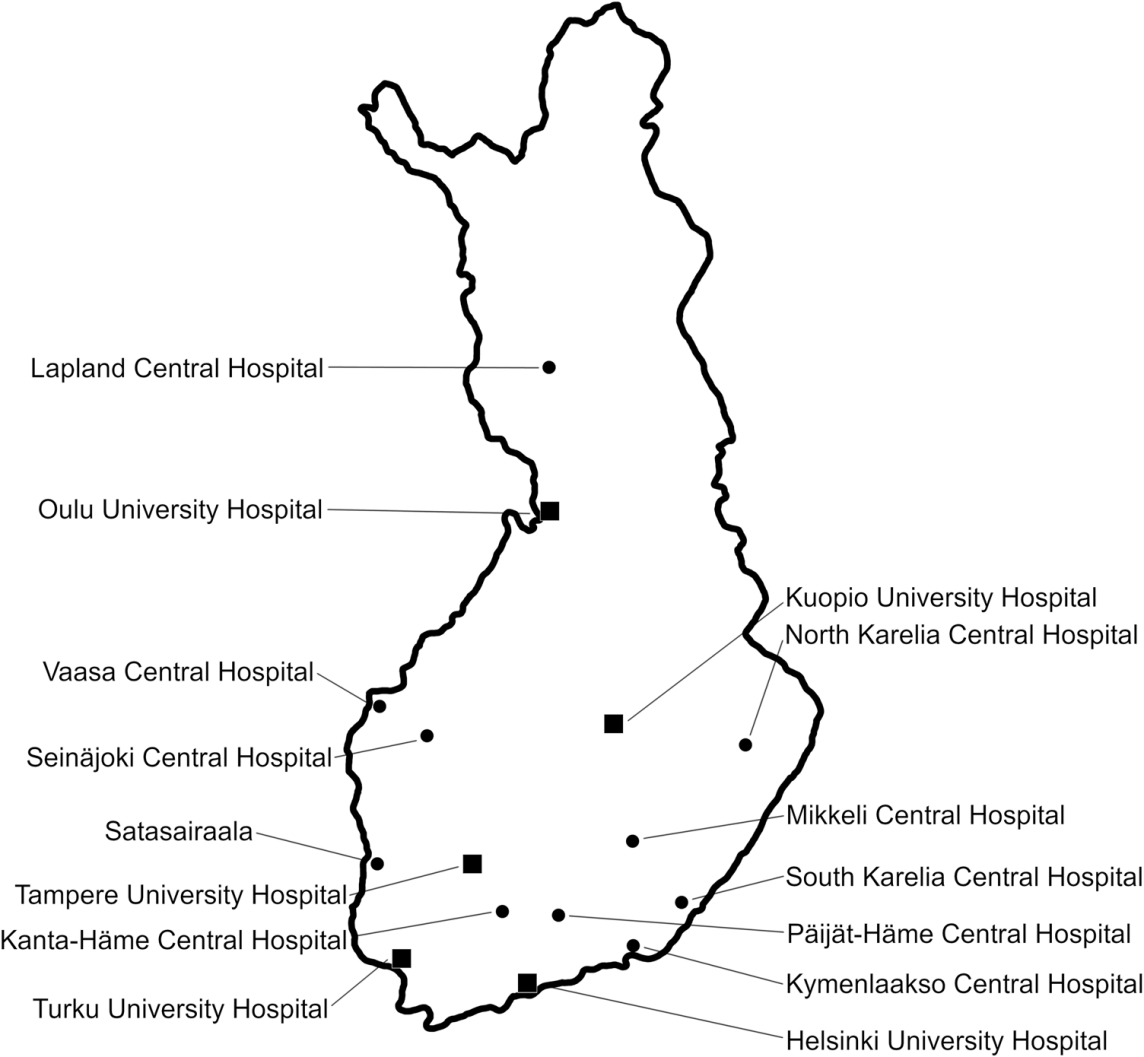
The geographic distribution of participating hospitals

A retrospective non-interventional case series study using real-world data was conducted. All but one Finnish hospital where alemtuzumab had been administered to MS patients participated. The non-participating hospital had two alemtuzumab-treated patients. The participating hospitals included all five university hospitals of Finland and ten central hospitals (Fig. 1), making the data virtually nationwide. An institutional approval was obtained from each organization. A Research Ethics Committee approval or patient consent was not required.

All patients with a diagnosis of MS (ICD-8, -9, or -10) who had received alemtuzumab were included. Data were systematically acquired from electronic patient files during 2018–2019. This was done by the actual treating physician in a majority of hospitals. The timing of data cutoff varied between hospitals due to different schedules in approval processes. Whenever available, the following variables were collected: age at diagnosis and at treatment initiation; sex category; time of diagnosis; pre-existing comorbidities; smoking status; use of vitamin D, course of the disease at treatment initiation; Expanded Disability Status Scale (EDSS) at treatment initiation; DMTs before and after alemtuzumab; number and timing of alemtuzumab infusions; AEs; and outcomes of AEs. When calculating the total number of previous DMTs, subcutaneous interferons were grouped as one therapy. Efficacy was not assessed in this study.

### Adverse events

AEs were classified as either IARs or other AEs. Lymphopenia was not considered an AE, as it represents the therapeutic effect of alemtuzumab [10]. Lower urinary tract and upper respiratory tract infections were not considered AEs, as significant underreporting can be expected in these potentially self-limiting conditions. Relapses during follow-up were not considered as AEs, but neurological symptoms during IARs were documented. Serious adverse events (SAEs) were defined the same way as in the alemtuzumab clinical trials [29] as life-threatening, resulting in death, requiring or prolonging hospitalization, disabling, resulting in a congenital anomaly, or requiring intervention to prevent one of these outcomes.

The timing of an AE was defined as the day of first symptom manifestation, or when the AE was first recognized by a health care professional. An IAR was defined as a new symptom or finding presenting within 24 h after an infusion of alemtuzumab. However, urticaria or rash was classified as an IAR as long as it manifested within 48 h after the last dose. If it was obvious that a condition had developed over a long period of time, it was not classified as an IAR even if its first manifestation occurred after an infusion of alemtuzumab (e.g. corticosteroid-induced hyperglycemia in a patient with previously unidentified type 2 diabetes). To avoid fragmentation of study data, all infections and cases of AAC were analyzed as other AE whether or not they presented within 24 h after an infusion.

### Statistics

Descriptive analysis was performed on pseudo-anonymized data using IBM SPSS Statistics 25. Incidence rates of AEs were calculated using Stata 16.0. Numerical variables were expressed as means with standard deviations (SDs) or medians with interquartile ranges (IQRs). Categorical variables were expressed as frequencies and proportions. Missing months were imputed as July, and missing days were imputed as the 15th day of the month. Follow-up time was calculated from the first infusion of alemtuzumab to data acquisition, death, or patient being lost to follow-up (i.e. moving to a location wherefrom data was inaccessible). When calculating incidences of IARs, multiple symptoms or findings documented during the same course were calculated as one IAR. A Mantel–Haenszel-type method was used to determine whether pre-existing autoimmune diseases were associated with a higher incidence of secondary autoimmunity.

## Results

### Study sample

The study sample included data of 121 MS patients who had received alemtuzumab during 2013–2019 (Table 1). Median follow-up time after treatment initiation was 30.3 months (IQR 20.9–42.5 months). Follow-up exceeded 12 months in 108 patients (89.3%), and 24 months in 78 patients (64.5%). A majority of patients had received previous DMTs prior to alemtuzumab (*n* = 100, 82.6%). The most common last preceding therapies were fingolimod (*n* = 49, 40.5%) and natalizumab (*n* = 20, 16.5%). Although alemtuzumab is only indicated for the treatment of RRMS, we identified two patients whose course of the disease was reported to be secondary-progressive multiple sclerosis (SPMS) at the time of alemtuzumab initiation. However, these patients still had relapses at treatment initiation, and therefore, were progressing with activity.

**Table 1:** Demographic details and baseline characteristics of the study sample

	*n*	%	
Sex category			
Female	90	74.4	
Male	31	25.6	
Course of disease			
Relapsive-remitting MS	119	98.3	
Secondary-progressive MS	2	1.7	
Number of previous DMTs			
0	21	17.4	
1	19	15.7	
2	22	18.2	
3	27	22.3	
4	23	19.0	
5	9	7.4	
Previously treated malignancy	1	0.8	
Reported use of vitamin D during alemtuzumab	89	73.6	
Reported (any amount of) smoking during alemtuzumab	27	22.3	
	Median	IQR	Range
Age at diagnosis of MS, years	26.6	22.2–32.3	13.8–59.2
Age at initiation of alemtuzumab, years	32.0	28.3–37.8	18.2–59.3
EDSS at initiation of alemtuzumab	3.0	2.0–5.0	0–8.0
Time from diagnosis to initiation of alemtuzumab, years	5.3	1.1–8.5	0.1–23.5
Time from discontinuation of previous DMT, months	2.1	1.6–3.4	0–38.2

*MS* multiple sclerosis, *DMT* disease-modifying therapy, *IQR* interquartile range, *EDSS* Expanded Disability Status Scale

**Fig. 2:**
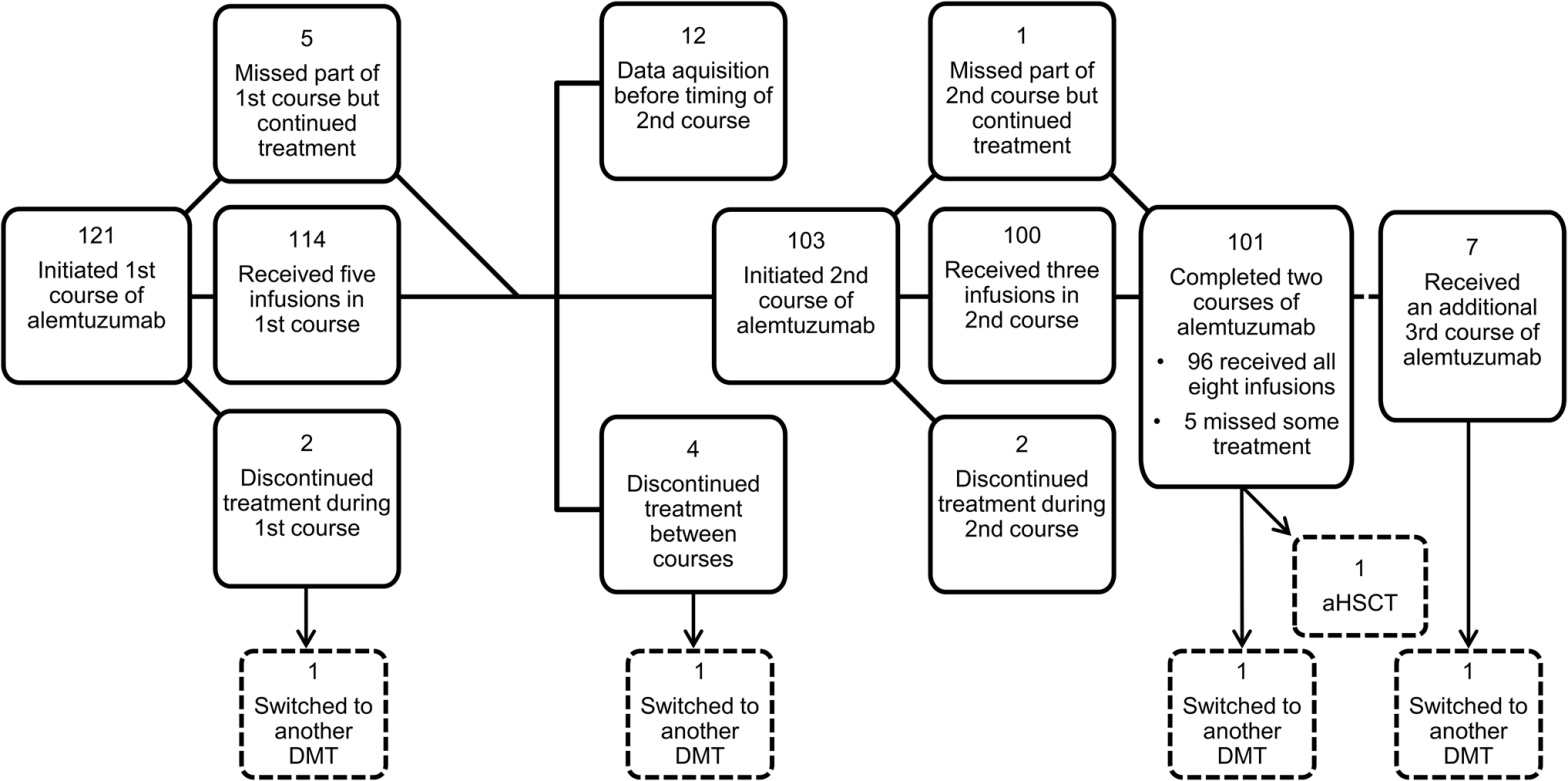
A flow chart displaying patients receiving treatment during each course of alemtuzumab. *aHSCT* autologous hematopoietic stem-cell transplantation, *DMT* disease-modifying therapy

All treatment courses were administered according to the label using the standard dose of 12 mg daily. At data acquisition, two complete courses of alemtuzumab had been administered to altogether 96 patients (79.3%, Fig. 2). In addition, 5 patients (4.1%) had received alemtuzumab during two courses, but missed part of their treatment due to AEs. A third course had been administered to 7 patients (5.8%) due to disease activity. Other subsequent DMTs were initiated in 4 patients (3.3%) after alemtuzumab (cladribine, daclizumab, dimethyl fumarate, and ocrelizumab). In addition, 1 patient underwent autologous hematopoietic stem-cell transplantation after receiving two courses of alemtuzumab.

### Adverse events

The incidences of AEs are presented by category in Tables 2 and 3. Discontinuation occurred in six patients due to AEs (5.0%), and two patients due to lymphopenia (1.7%), as presented in Table 4. One patient died of HLH, as also described in a case report published earlier [17]. One patient with severe disability died of pneumonia three years after the last infusion of alemtuzumab with MS being the underlying cause of death. Five patients (4.1%) had no AEs.

**Table 2:** Incidences of infusion-associated reactions according to treatment course

	1st course		2nd course		3rd course	
	*n*	%	*n*	%	*n*	%
Any infusion-associated reaction	102	84.3	59	57.3	4	57.1
Urticaria or rash	63	52.1	22	21.3	1	14.3
Headache	22	18.2	20	19.4	1	14.3
Hyperthermia or fever	18	14.9	13	12.6	2	28.6
Alterations in heart rate or palpitations	21	17.4	9	8.7	0	
Neurological symptoms	19	15.7	8	7.8	0	
Serious infusion-associated reaction	13	10.7	3	2.9	0	
Patients receiving alemtuzumab in each course	121		103		7	

Only the most frequently observed symptoms or findings are presented separately

**Table 3:** Incidence rates of adverse events of interest

	Patients with event		Incidence rate
	*n*	%	Number of events per 100 patient-years
Any AE	116	95.9	
Any IAR	109	90.1	
Any AE excluding IARs	65	53.7	33.1
Any AE leading to treatment discontinuation	6^a^	5.0	
Any serious AE	39	32.2	
Any serious IAR	15	12.4	
Any SAE excluding IARs	28	23.1	10.2
Death	2	1.7	0.6
Any infection event	30	24.8	11.8
Serious infection event	10	8.3	3.4
Any autoimmune AE	37	30.6	13.8
Autoimmune thyroid event	32	26.4	11.8
Serious autoimmune thyroid event	5	4.1	1.6
ITP	2	1.7	0.6
Acute acalculous cholecystitis	3	2.5	1.0
Malignant disease	4	3.3	1.3

*AE* adverse event, *IAR* infusion-associated reaction, *SAE* serious adverse event, *ITP* immune thrombocytopenic purpura

^a^In addition, two patients (1.7%) discontinued due to lymphopenia, which we did not regard as an adverse event

**Table 4:** Reasons for discontinuation of treatment

	*n*
Adverse events resulting in discontinuation	
Acute acalculous cholecystitis	2
Hepatic or hepatobiliary reaction	2
Pulmonary reaction with edema	1
Pyelonephritis	1
Other reasons for discontinuation	
Acute lymphopenia	1
Prolonged lymphopenia	1

### Infusion-associated reactions

IARs were documented in 84.3%, 57.3%, and 57.1% of patients during treatment courses 1–3, respectively (Table 2). The most frequently observed symptoms or findings of an IAR were urticaria or rash, headache, and hyperthermia or fever. Most of the serious IARs occurred during the first course. One patient experienced a serious IAR in both courses. No discontinuations were reported due to IARs, although four patients discontinued treatment during a course of alemtuzumab due to events other than IARs (AAC, acute lymphopenia, and pyelonephritis).

Of the 15 patients who continued to receive a second course after not having an IAR in their first course, 8 patients (53.3%) did not have an IAR on the second course either. It is noteworthy that although an IAR was documented in altogether 165 treatment courses (out of 231 treatment courses administered), 39% of them did not require any extra interventions, examinations, prolonged hospitalization, or re-hospitalization.

Neurological symptoms were documented during 27 IARs, occurring in 11.7% of all courses administered. They were mostly exacerbations of pre-existing neurological symptoms but also new neurological symptoms were documented. Of the 16 patients who experienced a neurological symptom during their first course and continued to receive a second course, only 4 patients (25%) had a neurological symptom during the second course as well.

### Other adverse events

#### Infections

Infections were observed in 30 patients (24.8%; Table 3). *Herpes zoster* reactivation was the most frequent infection (10 patients, 3.4/100 patient-years). Serious infections occurred in 10 patients, with pneumonia being the most common serious infection (4 patients, 1.3/100 patient-years). Other serious infections included cases of *herpes zoster*, pyelonephritis, dental infection, and unspecified infection with a strong suspicion of bacterial etiology. One patient discontinued treatment due to pyelonephritis, which interrupted the second treatment course. No cases of *Listeria monocytogenes* were observed.

#### Secondary autoimmunity

**Fig. 3:**
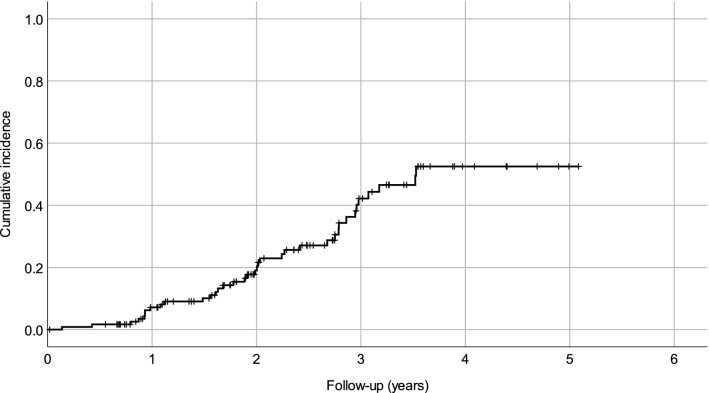
A survival curve displaying the cumulative incidence of first autoimmune adverse event

Autoimmune AEs were observed in altogether 37 patients (30.6%; Table 3; Fig. 3), most of whom developed autoimmune thyroid AEs. The most frequent thyroid AE was hyperthyroidism. Serious autoimmune thyroid AEs included four cases of hyperthyroidism resulting in thyroidectomy and one case of thyroiditis resulting in hospitalization.

Two cases of ITP were observed, one of which was serious. In addition, single cases of the following autoimmune AEs were observed: asthma; HLH, psoriasis; thrombotic thrombocytopenic purpura (TTP); T1D; and vitiligo. Of these, the cases with HLH, TTP, and T1D were classified as serious. The patient with TTP required intensive care and plasmapheresis.

Of the 37 patients who developed autoimmune AEs, 5 had a pre-existing autoimmune disease in addition to MS. However, having a pre-existing autoimmune disease was not associated with an increased incidence of secondary autoimmunity (*p* = 0.95). Three patients developed more than one new autoimmune disease after alemtuzumab initiation (multiple thyroid AEs in single patients were only counted as one). One patient suffered from recurrent arthralgia and myalgia which was not classified as an autoimmune disease even though the patient recovered after steroid treatment, as no evidence of an underlying rheumatic disease could be found. No cases of acquired hemophilia A, anti-glomerular basement membrane nephropathy, or autoimmune hepatitis were observed.

#### Hepatobiliary adverse events

AAC was observed in three patients (2.5%). In two of the cases, AAC manifested during or after the third infusion of the first course of treatment, and resulted in discontinuation of treatment. The third case manifested one year after the second course of treatment in a patient who soon after developed HLH. In addition, one case of acute calculous cholecystitis resulting in cholecystectomy was observed. All cases of cholecystitis were serious.

Two patients developed unspecified reactions with either hepatic or hepatobiliary involvement. The first case had elevated alanine aminotransferase (ALT), and was suspected for autoimmune hepatitis after responding to steroid treatment. The second case had both elevated ALT and amylase together with mild dilatation of bile ducts in an ultrasonography and eosinophilic granulocytes in a liver biopsy. Drug reaction or cholangitis was suspected. A definitive diagnosis could not be made in either case. Both AEs were classified as serious, and resulted in discontinuation of treatment, as they manifested between treatment courses.

#### Neoplasms

Malignancies were observed in four patients (3.3%). They included two cases of breast cancer, one cervical cancer, and one cervical carcinoma in situ. Additionally, one patient underwent a gynecological intervention due to cervical dysplasia, which was classified as a precancerous condition. No cancer-related deaths were observed during the studied period.

#### Unclassified adverse events

Various other AEs were also observed in the study data, mostly in single patients. A few of these AEs were considered to be of particular interest. One patient discontinued treatment after developing a pulmonary reaction with edema four days after completing the first course of treatment. The condition was diagnosed as a drug reaction to alemtuzumab, and treated with steroids by a pulmonologist. One patient developed sarcoidosis with both pulmonary and renal manifestations almost three years after the second course of alemtuzumab. The patient presented with acute symptoms and required urgent hospitalization due to hypercalcemia.

No cases of myocardial infarction were observed. One ischemic stroke was observed in a patient previously treated with plasmapheresis. In addition, one patient with ITP developed recurrent thrombophlebitis. No cases of autoimmune nephropathy were observed, but one patient with pre-existing type 2 diabetes developed mild nephropathy. Asymptomatic microalbuminuria and microhematuria were observed in one patient.

Two patients suffered from transient diarrhea requiring treatment in the emergency room 1 and 4 months after receiving alemtuzumab. In one of the cases, infectious colitis was suspected. A third patient with diarrhea and loss of appetite was evaluated in an outpatient setting. Despite investigations, an underlying pathology could not be identified.

## Discussion

In this large nationwide real-world cohort, we report the safety of alemtuzumab with a special focus on SAEs, secondary autoimmunity, and previously unreported AEs. The present study can be compared with five previous real-world studies evaluating the use of the currently recommended dose of alemtuzumab, as well as the 12 mg treatment arm of the pivotal CARE-MS II core trial, in which study patients had received previous DMTs (Table 5) [3, 30–34]. However, comparison between studies is not straightforward due to different study protocols and varying follow-up times. In this report, four additional studies in which at least some patients had received previous or off-label doses of alemtuzumab, as well as two studies not reporting the dose of alemtuzumab, were not included for comparison [35–40].

**Table 5:** Our results compared with previous findings from real-world studies evaluating the use of the currently recommended dose of alemtuzumab, as well as the 12 mg treatment arm of the pivotal CARE-MS II core trial

Reference	Present study	Huhn et al	Prosperini et al	Frau et al	Zmira et al	Brecl Jakob et al	CARE-MS II (12 mg group)
		[30]	[31]	[32]	[33]	[34]	[3, 5]
*N*	121	50	40	90	35	71	426
Follow-up time, months	30 (median)	15 (mean)	36	27 (mean)	24	38 (mean)	24 (core trial)
Females, %	74.4	60.0	82.5	74.4	54.3	71.8	66
Previous DMT use, %	82.6	100	97.5	92.2	100	71.8	100
EDSS at treatment initiation, median	3.0	3.0	4.0	2.5	4.0	3.0	2.5
Any AE, %	95.9	NA	NA	NA	100	84.1	98
Any IAR	90.1	NA	95	95.5	100	59.2	90
Any non-IAR AE *(per 100 PY)*	53.7 *(33.1)*	NA	37.5	NA	37.1	NA	NA *(255.8)*
Any SAE, % *﻿(per 100 PY)*	32.2	NA	9 events^a^	NA	NA	NA	13% *(11.1)*^b^
Any serious IAR	12.4	NA	7 events^a^	NA	NA	NA	3%
Any non-IAR SAE *(per 100 PY)*	23.1 *(10.2)*	NA	2 events^a^	NA	NA	NA	NA *(10.0)*
Autoimmune AE, %	30.6	4	9 events^a^	12.2	8.6	NA	NA
Autoimmune thyroid AE *(per 100 PY)*^c^	26.4 *(11.8)*	2	8 events^a^	11.1	8.6	31.9	16 *(8.8)*^c^
ITP *(per 100 PY)*	1.7 *(0.6)*	0	0	3.3	0	1.4	1 *(0.5)*
Other autoimmune AEs	Asthma, HLH, psoriasis, TTP, T1D, vitiligo	Hemolytic anemia	Acquired hemophilia A	–	–	–	Hemolytic anemia, membranous nephropathy
Serious infection, % *(per 100 PY)*	8.3 *(3.4)*	2	0	NA	NA	NA	4 *(1.9)*
Malignant disease, % *(per 100 PY)*	3.3 *(1.3)*	2	0	0	2.9	0	< 1 *(0.2)*

Values represent the proportions of patients with an AE. Incidence rates are provided for specific categories in parenthesis for the present study and the CARE-MS II trial

*AE* adverse event, *DMT* disease-modifying therapy, *EDSS *expanded disability status scale, *NA* not available, *IAR* infusion-associated reaction, *PY* patient-years, *SAE* serious adverse event, *ITP* immune thrombocytopenic purpura, *HLH* hemophagocytic lymphohistiocytosis, *TTP* thrombotic thrombocytopenic purpura, *T1D* type 1 diabetes

^a^Only the number of events is provided, and not the proportion of patients with an event

^b^Excluding multiple sclerosis relapses

^c^In the CARE-MS II trial, the reported value represents “any thyroid event”, and not “any autoimmune thyroid event”

SAEs—serious IARs in particular—were more frequent in this cohort than in the CARE-MS II core trial (Table 5) [3]. The slightly longer follow-up time in the present study is not likely to explain this difference, as a large amount of SAEs in this cohort were serious IARs. In our cohort, serious IARs most often manifested during the first course of alemtuzumab even though premedication with intravenous steroids was administered. Also, serious infections and malignancies were observed at slightly higher incidence rates in this cohort when compared to the CARE-MS II core trial [5]. These differences may reflect different study populations, as real-world patients tend to have more comorbidities than patients in clinical trials. In the present study, we used the same definition for SAEs as used in clinical trials. Therefore, our findings should be comparable despite different settings.

It seems that AE severity is seldom assessed in retrospective studies. Only one large Italian cohort study reported seven serious IARs and two other SAEs during a 36-month follow-up of 40 MS patients [31]. Although they did not report incidences, their findings regarding serious IARs are similar to ours. This suggests that serious IARs are more frequent in a real-world setting in contrast to clinical studies, possibly reflecting higher comorbidity in real-world patients.

Autoimmune AEs were observed in 30.6% of patients in our study. The incidence rate of thyroid AEs in the present study was comparable to the CARE-MS II core trial (Table 5), although slightly different AE categories were used: our study reports the incidence rate of autoimmune thyroid AEs, whereas the CARE-MS II trial reported the incidence rate of all thyroid AEs including conditions such as goiter [3, 5]. In a recent real-world study by Brecl Jakob et al., more autoimmune thyroid AEs (31.9%) were observed than in the present study (26.4%), which may be explained by the longer follow-up time [34]. Other previous real-world studies have reported autoimmune thyroid AEs occurring in only 2–11% of patients, but these studies have had shorter follow-up times (Table 5) [30, 32, 33].

In addition to autoimmune thyroid AEs, two cases of ITP and six cases of other autoimmune AEs were observed, including one case of fatal HLH [17] and one case of TTP, which is a previously unreported autoimmune AE after alemtuzumab therapy. We hypothesize that either environmental factors or genetic predisposition to autoimmune diseases in the population may explain why so many different types of autoimmune AEs were observed in this cohort, as Finland is known to be a high-risk region for certain autoimmune diseases such as MS, T1D and coeliac disease [23, 24, 26, 27]. However, we did not observe any association between pre-existing autoimmune diseases and the development of secondary autoimmunity. Another possible explanation may be the previous exposure to different DMTs, which could modify long term immune responses. Only 17.4% of our patients were treatment-naive, whereas 48.7% of patients had received three or more previous DMTs.

We confirmed the findings of previous reports stating that cases of AAC, acute sarcoidosis, and T1D can be observed after alemtuzumab therapy for MS, highlighting the meaningfulness of post-marketing real-world studies [18, 41, 42]. Notably, reports of new AEs led to EMA initiating its review of alemtuzumab in 2019 [21]. In our cohort, one serious pulmonary reaction and two hepatic or hepatobiliary reactions were also observed, all of which led to treatment discontinuation. The various presentations of AEs in the present study emphasize the importance of collaboration with other medical specialties when diagnosing potential AEs after alemtuzumab therapy. Although malignancies were rare in our cohort, the occurrence of one cervical cancer, one cervical carcinoma in situ, and one cervical dysplasia underline the need for cervical screening.

IARs were common in the present study, which is in line with most previous studies (Table 5) [5, 31–33]. Only five patients (4.1%) missed part of their treatment due to an IAR, and the four discontinuations occurring during a course of alemtuzumab were due to events other than IARs. In general, it can be concluded that a majority of IARs are either mild or moderate in severity, and manageable in an inpatient setting. Furthermore, the incidence of IARs declined after the first course of alemtuzumab, and serious IARs were rare in subsequent courses.

The strength of the present study is the large sample size and the inclusion of almost every alemtuzumab-treated MS patient in Finland with both urban and rural areas covered. Finland is considered a genetically isolated nation [25]. Therefore, the present study had the advantage of evaluating the risk of secondary autoimmunity in a unique setting in contrast to other populations. Our cohort is the largest among real-world studies evaluating the use of the currently recommended dose of alemtuzumab for MS, and the first to present incidences of SAEs (Table 5). Data were collected systematically from comprehensive medical records, and in most cases this was done by the actual treating physician. Limitations to the present study include its retrospective setting and the lack of comparison to patients treated with other DMTs. A longer follow-up may reveal additional AEs and confirm the outcomes of some SAEs such as malignancies.

In conclusion, we observed more SAEs—serious IARs in particular—than in the comparable 12 mg treatment arm of the pivotal CARE-MS II trial [3]. In addition, we observed a previously unreported case of TTP. Even though alemtuzumab is a highly effective therapy for MS, vigorous monitoring with a long enough follow-up time is advised. Clinicians must be alert, as AEs are shown to present with various clinical manifestations. More nationwide cohort studies evaluating the safety of alemtuzumab are needed to identify the role of different ethnic and genetic backgrounds in the appearance of AEs.

## Data Availability

Research data are not shared due to privacy restrictions.
